# Special Issue “Applications of Machine Learning in Sports Medicine, Physical Activity, Posture, and Rehabilitation”

**DOI:** 10.3390/jfmk11010007

**Published:** 2025-12-25

**Authors:** Federico Roggio

**Affiliations:** Department of Biomedical and Biotechnological Sciences, Anatomy, Histology and Movement Sciences Section, School of Medicine, University of Catania, Via S. Sofia 87, 95123 Catania, Italy; federico.roggio@unict.it

## 1. Introduction

In recent years, machine learning (ML) has emerged as one of the most influential methodological advances in sports medicine, physical activity, posture assessment, and rehabilitation. The increasing availability of biomechanical, physiological, and behavioral data has created unprecedented opportunities to model human movement in a more comprehensive and individualized manner [1]. ML techniques have shown potential to improve performance analysis, injury risk assessment, rehabilitation monitoring, and the personalization of exercise programs, contributing to more proactive and data-informed decision-making processes [2]. However, alongside this rapid expansion, a critical issue has emerged: the gap between methodological sophistication and real-world applicability. High predictive accuracy alone does not guarantee clinical relevance, interpretability, or practical adoption [3]. In domains such as sports medicine and rehabilitation, ML models must be biologically coherent, transparent, and feasible in applied settings, often characterized by small datasets, heterogeneous populations, and contextual constraints. Recent literature has increasingly emphasized the importance of explainable models, low-cost sensing solutions, and ecologically valid validation protocols to ensure meaningful translation into practice [4].

Within this context, the present Special Issue brings together complementary contributions that collectively explore how ML can be effectively integrated into the study and application of human movement, spanning preventive health, performance analysis, gait and posture assessment, rehabilitation, and youth sport monitoring [5]. Rather than promoting ML as a universal solution, these studies critically examine both its strengths and its current limitations, offering a balanced and realistic perspective on its role within functional morphology and kinesiology.

A central theme emerging from this Special Issue is that machine learning becomes most effective when it is grounded in human physiology and progressively applied to increasingly complex movement scenarios, rather than being treated as a purely algorithmic solution. Kurokawa et al. (contribution 2), provided a solid physiological framework linking respiratory function, trunk muscle strength, and motor performance in a large clinical cohort. This study establishes clinically and biomechanically coherent relationships that are essential for the development of reliable data-driven models in rehabilitation. It highlights a key principle underlying the entire Special Issue, effective ML applications must be constrained and informed by well-characterized human physiology, rather than relying on abstract, data-driven associations alone.

Building on this physiological foundation, Balampanos et al. (contribution 6) demonstrated how interpretable ML models applied to accessible and low-cost physiological data can support early screening strategies in health-related contexts. Using raw bioelectrical impedance analysis data, the authors successfully classify osteopenia in perimenopausal women while maintaining transparency and physiological interpretability. This contribution exemplifies how ML can enhance preventive and community-based healthcare by extracting clinically meaningful information without replacing gold-standard diagnostics, reinforcing the value of explainable and scalable solutions.

Moving from health screening to functional movement assessment, Kokkotis et al. (contribution 5) addressed fall risk detection through gait analysis, integrating deep learning architectures with explainability techniques. Their study shows that, even in small-sample scenarios typical of clinical settings, ML models can achieve robust performance when supported by appropriate data representations. Crucially, the use of Grad-CAM enables the interpretation of model decisions in relation to specific gait phases and stability-related features, reinforcing the necessity of linking algorithmic outputs to biomechanical meaning in preventive and rehabilitative applications.

The transition from clinical movement analysis to sport-specific performance is represented by the study of Caprioli et al. (contribution 4), which introduced an innovative acoustic-based ML framework for assessing temporal and rhythmic components of tennis performance. By demonstrating strong agreement between ML-derived acoustic metrics and video-based ground truth, this work highlights the potential of alternative data modalities to provide ecologically valid, field-based assessments. It illustrates how ML can transform traditionally qualitative aspects of sport performance into objective, quantifiable indicators without increasing measurement invasiveness or cost.

Addressing performance in even more complex and continuous motor tasks, Chesher et al. (contribution 3) proposed a wearable-based ML framework capable of detecting cadence and task transitions across swimming, cycling, and running in triathlons. The high accuracy achieved across disciplines, combined with spatial and temporal visualization of performance fluctuations, demonstrates how ML can support the analysis of multi-discipline endurance performance in real competition environments. Equally important is the authors’ transparent discussion of task-dependent limitations, which reinforces the importance of ecological validity and contextual awareness when applying ML models in applied sport settings.

Finally, Teixeira et al. (contribution 1) addressed one of the most critical and often underreported aspects of applied machine learning: its limitations in real-world sport data. Focusing on youth football training load, the authors show that while maturation-related variables meaningfully contribute to performance variability, increasingly complex ML regressors do not necessarily improve predictive accuracy. This contribution plays a key editorial role by demonstrating that ML is not inherently superior to simpler, interpretable models, and that contextual, tactical, and task-related factors must be integrated to achieve meaningful and actionable predictions. Such methodological honesty strengthens the overall narrative of the Special Issue and provides a realistic framework for future developments.

## 2. Conclusions

Collectively, the contributions of this Special Issue demonstrate that ML has reached a level of maturity where its value lies not in novelty, but in integration. Across health screening, gait analysis, sport performance, rehabilitation, and youth training monitoring, ML proves most effective when it is interpretable, biologically coherent, and embedded within the realities of applied practice.

Looking forward, several key directions emerge. First, future research should prioritize explainable and hybrid modeling approaches, combining data-driven techniques with biomechanical and physiological knowledge. Second, greater emphasis should be placed on ecological validity, ensuring that models trained in controlled conditions remain robust in real-world environments. Third, multimodal data integration, linking kinematics, kinetics, physiological signals, and contextual information, represents a critical step toward more comprehensive representations of human movement.

At the same time, it highlights the need for methodological restraint. Not all movement-related problems require complex ML solutions, and performance gains should always be weighed against interpretability, feasibility, and clinical relevance. Ultimately, the future of machine learning in sports medicine, physical activity, posture, and rehabilitation will depend not on algorithmic complexity, but on the ability of researchers and practitioners to ask the right questions, select appropriate models, and translate outputs into actionable knowledge that improves health, performance, and quality of life.

## References

[1] Dindorf C., Bartaguiz E., Gassmann F., Fröhlich M. (2024). Artificial Intelligence in Sports, Movement, and Health.

[2] Roggio F., Trovato B., Sortino M., Musumeci G. (2024). A comprehensive analysis of the machine learning pose estimation models used in human movement and posture analyses: A narrative review. Heliyon.

[3] Ennab M., McHeick H. (2024). Enhancing interpretability and accuracy of AI models in healthcare: A comprehensive review on challenges and future directions. Front. Robot. AI.

[4] Souaifi M., Dhahbi W., Jebabli N., Ceylan H.I., Boujabli M., Muntean R.I., Dergaa I. (2025). Artificial Intelligence in Sports Biomechanics: A Scoping Review on Wearable Technology, Motion Analysis, and Injury Prevention. Bioengineering.

[5] Mateus N., Abade E., Coutinho D., Gómez M., Peñas C.L., Sampaio J. (2024). Empowering the Sports Scientist with Artificial Intelligence in Training, Performance, and Health Management. Sensors.

